# Comparison of PGH_2 _binding site in prostaglandin synthases

**DOI:** 10.1186/1471-2105-11-S1-S51

**Published:** 2010-01-18

**Authors:** Padmapriya Paragi-Vedanthi, Mukesh Doble

**Affiliations:** 1 Department of Biotechnology, Indian Institute of Technology Madras, Chennai, 600036, India

## Abstract

**Background:**

Prostaglandin H_2 _(PGH_2_) is a common precursor for the synthesis of five different Prostanoids via specific Prostanoid Synthases. The binding of this substrate with these Synthases is not properly understood. Moreover, currently no crystal structure of complexes bound with PGH_2 _has been reported. Hence, understanding the interactions of PGH_2 _and characterizing its binding sites in these synthases is crucial for developing novel therapeutics based on these proteins as targets.

**Results:**

Shape and physico-chemical properties of the PGH_2 _binding sites of the four prostanoid synthases were analyzed and compared in order to understand the molecular basis of the specificity. This study provides models with predicted pockets for the binding of PGH_2 _with PGD, PGE, PGF and PGI Synthases. The results closely match with available experimental data. The comparison showed seven physico-chemical features that are common to the four PGH_2 _binding sites. However this common pattern is not statistically unique and is not specific enough to distinguish between proteins that can or cannot bind PGH_2_. A large scale search in ASTRAL data bank, a non redundant Protein Data Bank, for a similar pattern showed the uniqueness of each of the PGH_2 _binding site in these Synthases.

**Conclusion:**

The binding pockets in PGDS, PGES, PGFS and PGIS are unique and do not share significant commonality which can be characterized as a PGH_2 _binding site. Local comparison of these protein structures highlights a case of convergent evolution in analogous functional sites

## Background

The Cyclooxygenase (COX) pathway is an important part of the arachidonic acid (AA) metabolism, generating five primary prostanoids. The biosynthesis of these prostanoids involves a sequence of three-steps namely 1) Release of arachidonic acid from phospholipids by secretory, cytoplasmic or from both types of phospholipase A_2 _(sPLA2 and cPLA2), 2) Oxygenation of AA by COX enzymes to form prostaglandin endoperoxide H_2 _(PGH_2_), and 3) the subsequent conversion of PGH_2 _to Prostaglandin D_2 _(PGD_2_), Prostaglandin E_2 _(PGE_2_), Prostaglandin F_2α _(PGF_2α_), prostacyclin (PGI_2_), and Thromboxane T_2 _(TXA_2_) via seven specific synthases [1,2].

PGD Synthase is responsible for the production of PGD2 as an allergy or inflammation mediator in mast and Th2 cells [5]. There are 3 isoforms of PGE Synthase (PGES) namely microsomal PGE Synthase -1 (mPGES-1), microsomal PGE Synthase -2 (mPGES-2) and cytoplasmic PGE Synthase (cPGES) responsible for the production of PGE_2_, which is an ultimate mediator of pain and inflammation. PGE_2 _also plays a critical role in regulating renal function and facilitating reproduction [3]. Prostaglandin F_2α _produced from PGF Synthase (PGFS) is a hormone-like substance participating in a wide range of body functions including the contraction and relaxation of smooth muscle, the dilation and constriction of blood vessels, control of blood pressure, and modulation of inflammation. PGF_2α _is used for the induction of abortion, for evacuation of the uterus after a missed abortion [4]. PGI_2 _produced by PGIS, and TXA_2 _produced by TXAS, are critical for the maintenance of homeostasis in the vascular tissue [6,7]. Since these five synthases, are involved in various important biological processes, they are potential drug targets and drugs are already in the market for the inhibition of PGDS, PGFS, PGIS and TXAS. mPGES-1 is being sought after as a novel target to relieve pain and inflammation after the withdrawal of popular COX-2 inhibitors from the market [8].

Understanding the interactions of PGH_2 _with these synthases and characterizing their binding sites is crucial for developing novel drugs and also to check for cross reactivity. PGH_2 _is an unstable compound and there are no structures of synthases available in the Protein Data Bank (PDB) [9] with it. In this paper, the PGH_2 _binding sites in these proteins were predicted using the PatchDock algorithm [10]. The predicted binding sites were then compared using MultiBind [11], a multiple binding site alignment tool to look for common pattern which might help us to characterize a PGH_2 _binding site.

## Methods

### Protein structure

The crystal structure of four of the proteins namely PGDS, PGES, PGFS and PGIS are available in the PBD, while such a structure is not available for mPGES-1, cPGES and TXAS. For the purpose of docking studies the following structures were used: (i) PGDS - The structure of human hematopoietic prostaglandin D synthase complexed with HQL-79 (PDB: 2cvd, [12]). (ii) PGES - The structure of Microsomal prostaglandin E synthase type-2 (PDB: 1z9h, [13]). (iii)PGFS - The structure of prostaglandin F synathase containing bimatoprost (PDB: 2f38, [14]) and (iv) PGIS - The structure of human prostacyclin synthase in complex with inhibitor minoxidil (PDB: 3b6h, [15]).

### Docking

The dockings of PGH_2 _with these synthases were performed to predict the putative binding site in the proteins. Docking models are obtained using the PatchDock algorithm [10]. This software takes two molecules as input and computes the three-dimensional transformation of one of them with respect to the other with the goal of maximizing the surface shape complementarities and at the same time minimizing the number of steric clashes. Given two molecules, PatchDock first divides their surfaces into patches according to their surface shape, such as concave, convex, or flat. Then, it applies the Geometric Hashing algorithm to match the concave patches of one with the convex patches of the other protein and flat patches with flat patches and generates a set of candidate transformations. A set of scoring functions based on the shape complementarities and the atomic desolvation energy of the transformed complex is evaluated. Finally, redundant solutions are discarded by the application of a RMSD (root-mean-square deviation) clustering. This program is tested and shown to successfully predict protein interactions for many examples [16-19].

### Binding site alignment

The alignment of these predicted binding sites for PGH_2 _in the four synthases was performed using the MultiBind algorithm developed by Shulmana et. al [11].

This algorithm performs multiple alignments of the binding sites and recognizes the structurally conserved physicochemical and geometrical patterns that may be responsible for the binding. The physicochemical properties considered by the software are hydrophobic, aliphatic (ALI) and aromatic interactions (PII), hydrogen bond donors (DON), hydrogen bond acceptors (ACC), and mixed donor/acceptors (DAC). The algorithm finds a set of transformations which will superimpose the binding sites in a manner that will maximize the physicochemical score of the matched properties. This alignment between protein binding sites is performed even in the absence of overall sequence, fold, or binding partner similarity, and also it does not consider the location of the binding partners. The scoring function and the algorithm of MultiBind are described in detail elsewhere [11].

### Evaluation of common binding patterns

The frequency of random occurrence of structural patterns as recognized by MultiBind is searched with proteins in the ASTRAL dataset (V 1.73) [20,21]. This dataset consists of all known protein structures that have less than 40% sequence identity and hence it represents a non-redundant group. This dataset contains only the structures of PGDS and mPGES-2 that are known to bind to PGH_2_. The other two structures namely PGFS and PGIS were added to make the dataset of 7649 structures in which four of them were known to bind PGH_2_. The structural pattern was chosen from the pseudocenters of the first molecule in the input order. Each pattern that was recognized on the surface of some other protein was scored using the physicochemical scoring function of MultiBind as explained before. The frequency of occurrence of a pattern was calculated as the ratio between the numbers of times it was observed with a score higher than a reference score, relative to the total number of searched proteins. The reference score is defined to be the score of the outlier, i.e., lowest score of the most different binding site that participated in the pattern construction with MultiBind.(Example: If A, B, C and D are the protein compared, and the binding sites of A and D differ the most with the least MultiBind Score then that is taken as the reference score for comparisons of ABCD, ABD, ACD and AD with the ASTRAL dataset). The obtained ratio represents the estimation of the chances for a random occurrence of the recognized pattern. Using the score of the outlier as a reference score provides the highest possible ratio and the worst case estimation of the most frequent pattern [16]. The ratio of the number of similar patterns observed relative to the size of the searched dataset provides an estimation of the probability of observing such a pattern by chance, on a randomly selected protein. The lower the frequency of occurrence, rare is the pattern.

## Results

### Docking models of PGH_2 _with four synthases

PatchDock successfully detected the surface pockets of PGDS, PGES, PGFS and PGIS and they are in agreement with previously published data [22-30]on the putative binding site of PGH_2_. In all these analysis no apriori information was used as an input for the docking algorithms, i.e., the surface pockets on the receptor molecules were detected automatically.

**Figure 1:**
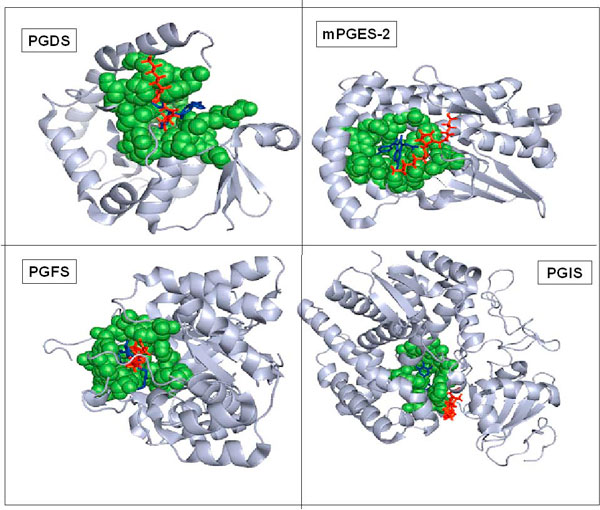
**Overlap of predicted PGH_2 _binding site with the ligand binding site in the four synthases**. The surface of the synthases are represented in cartoon and colored grey except the common active site residues represented in space-fill and are colored green. PGH_2 _is colored red, and the other ligands in the crystal structure (IMN, HQL-79, 15 M and MDX) are colored blue. The figure is prepared using PyMol [36]

For all the four synthases, out of the 20 docking solutions examined the putative binding site was predominantly located as the possible preferred binding pocket (in 16, 15, 15, 13 cases out of 20 solutions for PGFS, PGES, PGDS and PGIS respectively). A comparison between the predicted PGH_2 _binding site and the ligand binding site in crystal structures of these four structures suggested that in all the four cases the ligand bound in the crystal structure actually fits in the PGH_2 _binding pocket predicted in the current study. Based on residues extracted within 6Ǻ distance from the bound PGH2 and Ligand, it was found that common amino acid residues participated in the binding of PGH_2 _to the synthases in the docked model and the ligand in the crystal structure obtained from PDB Among the common residues, the cofactors GSH, NADP and HEME involved in the catalytic mechanism of the synthases PGDS (GSH) [12], PGFS (NADP) [14] and PGIS (HEME) [15] respectively are also shown in Figure 1.

### Binding site alignment and analysis

After obtaining the docking models and information about the PGH_2 _binding pocket, the goal was to compare these four predicted binding pockets and to determine the common features which facilitate the binding of the same substrate, PGH_2_.

The four binding sites compared here have different overall sequences and conserved residue patterns and are also structurally not related. Due to the above-mentioned differences, the proteins in this study cannot be aligned by standard alignment methods, that assume similarity of either sequence or backbone patterns. Thus, to compare between the predicted binding sites of the modelled complexes, we used MultiBind which performs a multiple structure alignment between protein binding sites, in the absence of overall sequence, fold, or binding partner similarity and recognizes the common spatial arrangements of physicochemical properties shared between the compared binding sites. The ligand from the crystal structures were used to extract the binding site pocket with the MULTIBIND algorithm rather with the PGH_2 _docked models as the algorithm do not accept docked models as input.

### Multiple alignment of all the four binding sites

**Figure 2:**
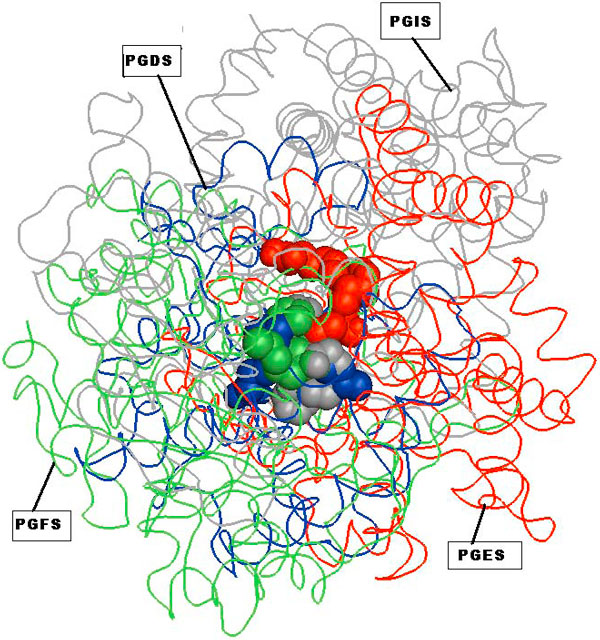
**Superimposition of the four synthases based on transformations suggested by MultiBind**. Spatial arrangement of the recognized features and the superimposition of the proteins and the PGH_2 _ligands, according to the transformations suggested by MultiBind. The structures of the four proteins are represented by strands. PGDS - blue, PGES - red, PGFS - green and PGIS - gray. PGH_2 _are represented as space fill and colored according to the protein. The ligand molecules are presented for verification purpose only and are not a part of the input to MultiBind. The figure is prepared using PyMol [36]

Alignment of all the four binding sites of PGH_2 _indicated a common pattern of seven physicochemical properties, namely one hydrogen bond acceptor/donor (DAC), three PII interactions, and three aliphatic interactions (ALI). Table 1 lists the residue numbers, residue types and the common physiochemical parameters identified for each of the four binding sites. Although the substrate PGH_2_, the ligand and its interactions with the protein were not taken into account during any of the computational steps performed by MultiBind, it never-the-less detected the key residues thought to be involved in the catalytic mechanism and superimposed the ligand molecules to similar locations in space, supporting the correctness of the alignment.(Figure 2). However this common pattern when searched for was found in 11% of the proteins in the ASTRAL dataset. This suggests that the detected pattern is not likely to be specific enough to distinguish between proteins that can and those that cannot bind PGH_2_.

**Table 1:** Details of the common pattern calculated between the four PGH2 binding sites using MultiBind. PII (aromatic) interactions, hydrogen bond acceptor (ACC), or mixed donor-acceptor (DON, DAC) and Aliphatic interactions(ALI).

**Site 1: PGES/**1z9hB			**Site 2: PGDS/**2cvdD			**Site 3:PGFS/**2f38A			**Site 4: PGIS/**3b6hA		
**Chain.ID**	**A. A**.	**Type**	**C** **hain.ID**	**A. A**.	**Type**	**Chain.ID**	**A. A**.	**Type**	**Chain.ID**	**A. A**.	**Type**

B.107	Tyr	PII	D.9	Phe	PII	A.24	Tyr	PII	A.99	Tyr	PII
B.109	Thr	PII	D.13	Gly	PII	A.117	His	PII	A.283	Ala	PII
B.109	Thr	ACC	D.13	Gly	ACC	A.117	His	DAC	A.283	Ala	ACC
B.110	Cys	ALI	D.14	Arg	ALI	A.54	Leu	ALI	A.283	Ala	ALI
B.246	Ile	ALI	D.99	Met	ALI	A.120	Met	ALI	A.128	Leu	ALI
B.250	Val	ALI	D.160	Leu	ALI	A.318	Pro	ALI	A.447	Ala	ALI
B.251	Tyr	PII	D.163	Phe	PII	A.319	Tyr	PII	A.434	Trp	PII

### Multiple alignments of three binding sites

The alignment between all the four binding sites resulted in a common pattern with seven physiochemical properties that is not unique enough for identification of the binding of PGH_2_. Therefore binding sites were aligned, eliminating one synthase at a time from the MultiBind during run time.

Comparison between three predicted binding sites of PGH_2 _at a time revealed a pattern of between 6-8 common physicochemical properties. The frequency of occurrence of this pattern in the ASTRAL dataset ranged between 17-38% showing that the frequency of occurrence of this pattern in this dataset is also too high to be statistically significant and once again it can be concluded that it is not specific enough to distinguish between proteins that can or cannot bind PGH_2_.

### Pairwise alignments

Pairwise surface alignments of the proteins detected more common features than those obtained while aligning three or four binding sites at a time. The summary of the six pairwise alignments of PGH_2 _binding sites is listed in Table 2. The two binding sites that were recognized to be most similar to each other are those of PGFS and PGIS. As can be seen from the similarity score, the binding site of the PGDS was the most different from all the rest. The number of common properties varies between 10 and 18. The occurrences of the common patterns based on the 6 pairwise alignments in the ASTRAL dataset ranges from 3 to 12% again indicating the uniqueness of each PGH_2 _binding site. This confirms that the PGH_2 _binding site of these four Synthases differ considerably and are also very different from any other binding pocket found on the proteins in the ASTRAL dataset.

**Table 2:** Pairwise alignments of PGH2 binding sites using MultiBind

Compared Proteins	No. of detected features	Score
PGFS-PGIS	18	41.2
PGDS-PGFS	13	38.0
PGES-PGIS	15	35.2
PGES-PGFS	12	33.7
PGES-PGDS	10	30.7
PGDS-PGIS	12	28.9

## Discussion

Based on the MultiBind similarity score, PGDS-PGFS is ranked second. PGFS is a dual acting enzyme leading to the formation of both PGF2α from PGH_2 _and 9α,11β-PGF2 (PGF2αβ) from PGD2. It can bind to both PGH_2 _and PGD_2 _[15]. The former is converted to PGD_2 _by PGDS; the later remains bound to the enzyme before being released. Having two common binding partners possibly explains their high similarity. It is surprising to note that both PGDS and PGES belonging to the same family with similar catalytic mechanism has the least number (11) of common features and are among the most different from each other when compared with other synthases. This is followed by PGIS-PGDS having 12 common features. mPGES-2 on the other hand is found to be more similar to PGIS which is a heme bound enzyme, than to PGDS. This can be explained based on a more recently solved structure in which PGES (PDBID: 2pbj[31]) is found to contain glutathione (GSH) and heme bound to it and it is involved in degradation reactions similar to that of cytochrome P450. It degrades PGH_2 _into 12(S)-hydroxy-5(Z), 8(E),10(E)-heptadecatrienoic acid and malondialdehyde rather than converting it to PGE_2 _[31].

**Figure 3:**
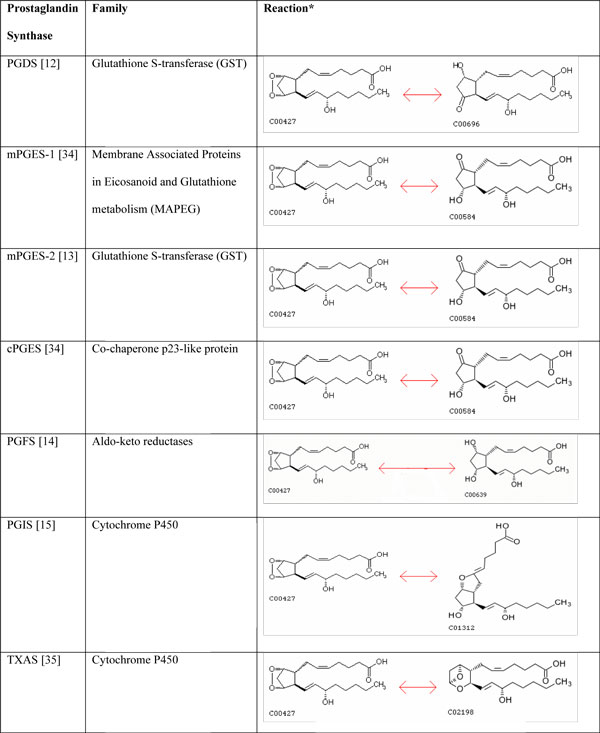
**Family and reaction details of the seven prostaglandin synthases**. *Reaction Schemes taken from the Kegg Database[37]

Interestingly all the seven Prostanoid synthases which bind to the same substrate PGH_2_, do not share any sequence identity amongst them, are structurally quite different and belong to different families (Figure 3). Yet they share PGH_2 _as the common binding partner. They even carry out the similar isomerization reaction at the cyclopentane ring of PGH_2_. A divergent evolutionary relation between PGDS and mPGES-2 and PGIS and TXAS might explain their specificity but in the other apparently disconnected families it is unlikely that divergent evolution would have played a role. Our comparison study shows that it is unlikely, that identical active site constellations are responsible for PGH_2 _specificity in these seven cases. Convergence seems to be limited to similarity in the ability to bind PGH_2 _specifically and may not extend to the precise way in which this is achieved as indicated by the lack of similarity which can be characterized as a PGH_2 _binding site. To answer these questions definitively we need much more biochemical information about each of the enzymes, details of the catalytic mechanism, rate constants, quantitative specificities, and regulatory dependencies.

But evolution of similar enzymatic function on different structural frameworks is not an entirely uncommon event. A classical example is that of serine proteases: the Ser-His-Asp triad is present in an almost identical three-dimensional constellation in the distinctly different structural frames of trypsin and subtilisin (and their relatives) [32,33]. Another example is each of the three families of sugar kinases which appear to have a distinct three-dimensional fold, and conserved sequence patterns are strikingly different for the three families. Yet each catalyzes chemically equivalent reactions on similar or identical substrates. The enzymatic function of sugar phosphorylation appears to have evolved independently on the three distinct structural frameworks, by convergent evolution[33]. Another aspect which needs consideration is that these proteins can rearrange and undergo conformational changes to accommodate the substrate. In practice, both the side-chains and the protein backbone can undergo conformational changes upon substrate binding. Even the PGH2 molecule with 14 torsional degrees of freedom of rotation is highly flexible and can fit into different active sites differently. Also both the docking and the alignment algorithms used in the current study considers rigid conformations and do not address the possibility of protein and substrate flexibility.

In summary, the Prostanoid Synthases present a remarkable diversity of specificities for the binding of PGH_2_. The discovery of this striking molecular dissimilarity, associated to a functional substrate similarity, may help in suggesting new experiments aimed at a deeper understanding of the cross reactivity of Prostanoid synthases known to be involved in many important biological processes and human diseases.

## Conclusion

A computational approach was employed to understand the interaction of PGH_2 _with the prostaglandin synthases. Docking models were consistent with the available experimental data for the interaction of PGH_2 _with the synthases. The spatial and physicochemical properties of the suggested binding sites were compared. A patterns common to all the four synthases was detected but it was not specific enough and was not likely to represent the features essential for the binding of PGH_2_. The alignment results suggest that the PGH_2 _binding sites are different on different proteins and they also have no close similarity with any other binding site found in the proteins of the ASTRAL dataset.

## Competing interests

The authors declare that they have no competing interests.

## Authors' contributions

PP conceived the study, carried out the major part of the work, analyzed the data and has written the first draft of the paper. MD participated in discussions and analysis of the study and helped drafting of the final manuscript.
